# Bias and Confounding in Studies of Chronic Mental Health Effects from Mefloquine Exposure

**DOI:** 10.4269/ajtmh.18-0771a

**Published:** 2019-02

**Authors:** Remington Lee Nevin

**Affiliations:** The Quinism Foundation; White River Junction, Vermont; E-mail: rnevin@quinism.org

Dear Sir,

I read with interest the article by Schneiderman et al.,^1^ which examined associations between self-reported mefloquine exposure and recent mental health symptoms among U.S. veterans enrolled in a population-based cohort study. Although the authors’ findings in unadjusted analysis reveal an increased risk that deployed veterans will screen positive for anxiety, depression, and posttraumatic stress disorder (PTSD)–like symptoms with mefloquine exposure, I am concerned that the magnitude of this observed association reflects the influence of information bias, specifically differential misclassification of a more appropriate exposure of interest. Similarly, I am concerned that the authors’ multivariate regression on covariates correlated with this exposure has resulted in unrecognized confounding. Consequently, I believe the authors’ conclusion that combat deployment exposure is a more significant predictor of subsequent mental health symptoms than the adverse effects of mefloquine is invalid and not supported by their data.

Mefloquine is postulated to cause chronic effects on mental health after an individual experiences certain acute adverse effects. Such adverse effects, which the manufacturer considers “prodromal,” are an indication of an individual’s susceptibility to a more serious event, particularly with continued use. According to the manufacturer’s current guidance, symptoms such as abnormal dreams and nightmares require the drug’s immediate discontinuation to reduce the risk of such symptoms persisting.^2^ According to a synthesis of recent data, abnormal dreams and nightmares lasting over 3 years after use may affect more than 2% of those exposed to mefloquine.^3^

Since mefloquine’s original U.S. licensing in 1989, the manufacturer has warned to discontinue the drug at the onset of symptoms of anxiety, depression, restlessness (i.e., insomnia), and confusion. In contrast to what is implied by the authors, the U.S. boxed warning was intended to direct attention to these and subsequently strengthened warnings, dating to 2002, to discontinue the drug at the onset of psychiatric symptoms, which can occur irrespective of a history of psychiatric illness.^4^ As a recent meta-analysis concludes, despite the fact that a considerable minority of those who use mefloquine experience prodromal symptoms, only 6% of users discontinue the drug as directed by the manufacturer.^5^ As suggested by the authors’ exclusion of this observation in their discussion, the significance of such continued symptomatic exposure among U.S. veterans remains widely overlooked.

As most of the individuals who do not experience prodromal symptoms with use of mefloquine are likely to be at low risk of chronic adverse mental health effects, their inclusion in the exposed group will result in differential exposure misclassification, which will bias unadjusted measures of association toward the null.

Conditions which increase the risk for continued exposure to the drug despite the onset of prodromal symptoms may mediate the development of subsequent chronic adverse mental health effects. Such conditions, including command-directed drug administration, may be correlated with combat exposures. As both causal symptomatic exposure to mefloquine and such combat exposures provide independent causal pathways to mental health symptoms assessed in the authors’ outcome measures,^3^ the authors’ multiple adjustments on combat deployment covariates without controlling for causal symptomatic exposure may result in intermediate confounding, unpredictably affecting observed associations.

Nevertheless, the authors’ findings from unadjusted analysis underscore concerns that chronic adverse mental health effects from mefloquine exposure may mimic those of combat deployment-related mental health conditions, and thus contribute to misdiagnosis.^6,7^ To assess causation, clinicians and researchers evaluating veterans with suggestive symptoms must take a careful mefloquine history,^8^ identifying when the symptoms first occurred in relation to drug use and to plausible traumatic events. For example, persistent symptoms of anxiety or nightmares, which developed before combat exposures, may be more plausibly due to mefloquine and should not contribute to a diagnosis of PTSD; as per current Diagnostic and Statistical Manual of Mental Disorders, Fifth Edition (DSM-5) criteria, the diagnosis is excluded if the condition is due to the effects of a medication.^9^ A medication-induced anxiety or sleep disorder secondary to mefloquine is a more appropriate diagnosis in these circumstances.

A standard instrument, the two-question White River Mefloquine Instrument (WRMI-2; Quinism Foundation, White River Junction, VT), has been proposed by our group to standardize ascertainment of mefloquine exposure and to encourage improved causality assessment, and it has been recommended for use within the U.S. Department of Veterans Affairs.^10^ We encourage researchers to use standardized methods such as this to assess causal exposure to mefloquine and to correctly control for this exposure in future analyses.
